# Influence of Quantum Dot Concentration on Carrier Transport in ZnO:TiO_2_ Nano-Hybrid Photoanodes for Quantum Dot-Sensitized Solar Cells

**DOI:** 10.3390/nano6110191

**Published:** 2016-10-25

**Authors:** Francis S. Maloney, Uma Poudyal, Weimin Chen, Wenyong Wang

**Affiliations:** Department of Physics and Astronomy, University of Wyoming, Laramie, WY 82071, USA; upoudyal@uwyo.edu (U.P.); wmchen@wit.edu.cn (W.C.); wwang5@uwyo.edu (W.W.)

**Keywords:** quantum dot sensitized solar cell, carrier transport, intensity modulated photovoltage spectroscopy

## Abstract

Zinc oxide nanowire and titanium dioxide nanoparticle (ZnO:TiO_2_ NW/NP) hybrid films were utilized as the photoanode layer in quantum dot-sensitized solar cells (QDSSCs). CdSe quantum dots (QDs) with a ZnS passivation layer were deposited on the ZnO:TiO_2_ NW/NP layer as a photosensitizer by successive ion layer adsorption and reaction (SILAR). Cells were fabricated using a solid-state polymer electrolyte and intensity-modulated photovoltage and photocurrent spectroscopy (IMVS/PS) was carried out to study the electron transport properties of the cell. Increasing the SILAR coating number enhanced the total charge collection efficiency of the cell. The electron transport time constant and diffusion length were found to decrease as more QD layers were added.

## 1. Introduction

Since the development of the dye-sensitized solar cells (DSSCs) in 1991 [1], both dye-sensitized and quantum dot sensitized solar cells (QDSSCs) have attracted enormous attention in alternative energy research in both academic and industrial laboratories. To convert sunlight into energy, sensitized solar cells make use of a semiconducting photoanode coated with a light absorbing layer. Upon absorption, this sensitizing layer will generate an exciton (electron-hole pair), leading to the injection of an electron to the photoanode, and a hole to the electrolyte. Quantum dots (QDs) can be used as sensitizers because they are generally small band-gap materials which can inject electrons into a large band-gap material such as TiO_2_ or ZnO [2,3]. Unlike molecular dyes, inorganic QDs represent the next-generation of solar cell sensitizers and have demonstrated many advantages over dyes including a size-dependent band-gap tunable to the infrared (IR) range, high stability and resistance to oxidation, possibilities for multi-layering of materials, an ability to generate multiple excitons, and the many permutations of materials that can comprise them. [4,5] QDs can also be grown directly onto a photoanode using a variety of techniques. Some methods include chemical bath depositions (CBD), pulsed laser deposition (PLD), spin-casting, dip-coating or successive ionic layer adsorption and reaction (SILAR) [5,6,7]. SILAR deposition has several advantages over these previous methods in that it allows easy control over the size and density of the QDs, since the dot synthesis takes place during the cycling. It is also relatively fast (the longest of depositions in this study lasting on the order of hours). SILAR is also considered the best method for depositing multilayers over QD cores by alternating the injection of cationic and anionic precursors [5].

QDs can be coupled with a variety of semiconducting photocatalysts to make QDSSCs. TiO_2_ has been the photocatalyst of choice for most DSSCs and QDSSCs due to its relatively low cost, low toxicity, chemical stability, and high efficiency in separating and transferring photogenerated charges to some other material interface [8]. Zinc oxide is another widely used photocatalyst with a similar band-gap to TiO_2_ (~3.2–3.4 eV at room temperature), though ZnO does not share the same chemical stability.

Another interesting alternative is the combination of the two oxides into a ZnO/TiO_2_ coupled polycrystalline system. Due to the low electron affinity and higher negativity of the ZnO conduction band edge compared to TiO_2_, electron-hole separation at the interface is facilitated and the carrier lifetime can be enhanced [9,10,11]. In the past, this system has been used as a photocatalytic reagent, where electron-hole pairs generated from light absorption and help trigger a redox reaction with a phenol or acid group adsorbed on the surface [11,12,13,14]. The same ZnO/TiO_2_ interface mechanism has also been used to reduce the recombination rate between the electrode and the electrolyte in DSCCS and QDSSCs [11,15]. Electrons in ZnO nanoparticles (NPs) or nanowires (NWs) can thereby be shielded from reacting with oxidized electrolyte species by an energy barrier established by the TiO_2_, causing an increase in carrier lifetime and an overall improvement in the performance of the device. Very few studies have utilized zinc oxide NWs fused with TiO_2_ NPs as the photoanode layer in DSSCs [16].

In this work, we studied charge transport in QDSSCs that utilize the zinc titanium oxide (ZnO:TiO_2_) hybrid nanostructures as the photoanode and a solid-state polymer electrolyte.

## 2. Results

**Figure 1:**
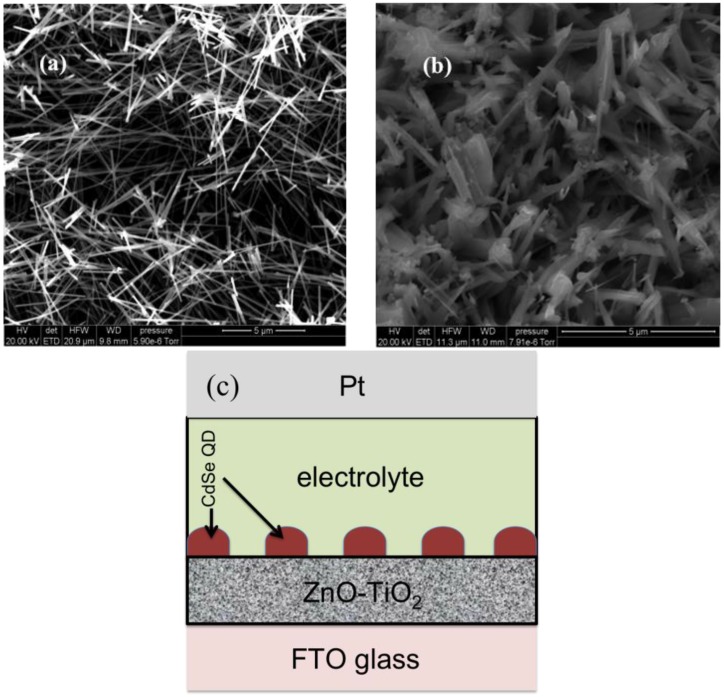
(**a**) Scanning electron microscopy (SEM) image of the as-grown ZnO nanowires. (**b**) Hybrid film after sintering with TiO_2_. (**c**) Schematic of cell structure.

Figure 1a shows the scanning electron microscopy (SEM) image of the as-grown ZnO nanowires, while Figure 1b shows the sample after sintering with TiO_2_. Before the TiO_2_ deposition, the average NW length was found to be ~10–15 μm and the average width was ~200 nm. After sintering, it can be seen that the TiO_2_ nanoparticles fused and spread mostly evenly over the surface of the NWs, however the width distribution greatly increased.

**Figure 2:**
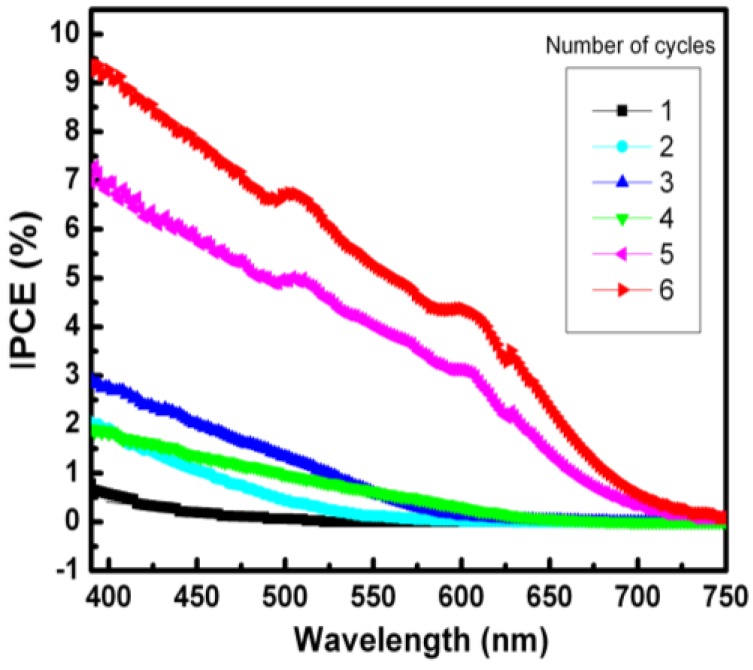
Incident-photon-to-electron-conversion efficiency (IPCE) signals as a function of increasing successive ion layer adsorption and reaction (SILAR) cycle number.

The diagram in Figure 1c shows the conceived schematic (not to scale) of the cell structure including the ZnO-TiO_2_ electrode with a QD coating, fluorine-doped tin oxide/platinum (FTO/Pt) electrode and polymer electrolyte. Incident-photon-to-electron-conversion efficiency (IPCE) measurements were performed on the finished cells and the results are shown in Figure 2. Devices coated with one through four cycles showed weak photocell performance subject to large device variations. At four or more cycles, the photovoltaic response becomes more prominent and indicates the limit of QD coverage necessary for efficient light harvesting and photo-generation of electrons. The spectra show a red-shift with increasing SILAR cycles, corresponding to the increase in the QD size and thus the reduction of the band-gap.

Short circuit currents (J_sc_) were estimated by integrating the area under the IPCE curves. These values were ~1.0, 10.3, 18.4, 14.5, 95.3 and 127.0 μA/cm^2^ for 1–6 cycles, respectively.

**Figure 3:**
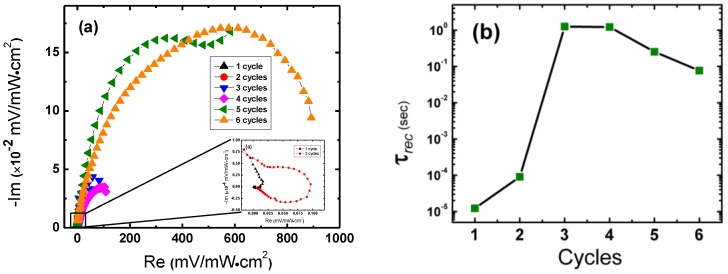
(**a**) Intensity modulated photovoltage spectroscopy (IMVS) Nyquist plot for ZnO:TiO_2_ nanowire photoanode coated with CdSe quantum dots (QDs) by increasing SILAR deposition cycles under 1 sun illumination. (**b**) Recombination time constants calculated from IMVS data.

Figure 3a shows the Nyquist diagram of the intensity modulated photovoltage spectra (IMVS) of the QDSSCs under 1 sun intensity for 1–6 SILAR cycle coatings. High to low frequency reads from left to right. The recombination and transport time constants (τ_rec_ and τ_trs_, respectively) can be derived using the equation

τ_rec_ (or τ_trs_) = 1/(2π*f*_min_) (1)

where *f*_min_ is the characteristic frequency taken from the minimum of the imaginary component of the spectrum [17]. The resultant τ_rec_ are shown in Figure 3b.

**Figure 4:**
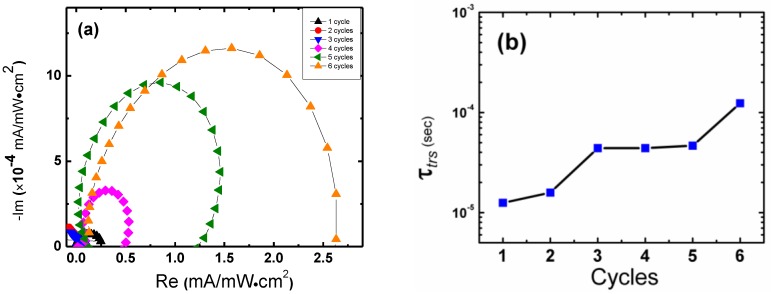
(**a**) Intensity modulated photocurrent spectroscopy (IMPS) Nyquist plot for ZnO:TiO_2_ nanowire photoanode coated with CdSe quantum dots (QDs) by increasing SILAR deposition cycles under 1 sun illumination. (**b**) Transport time constants, τ_trs,_ calculated from the IMPS data for 1 to 6 SILAR cycles.

Figure 4a shows the Nyquist plot of the intensity modulated photocurrent spectra (IMPS). Figure 4b shows the electron transport times, τ_trs_, as a function of SILAR cycle number. The diffusion coefficients, *D_n_*, and diffusion lengths, *L*_D_ were also examined. The value for *D_n_* was estimated using the equation

(2) $$D_{n}=\frac{d^{2}}{2.35\tau_{\mathrm{trs}}}$$

where *d* is the photoanode film thickness [18]. The electron diffusion length, *L*_D_, of each device was calculated using *D_n_*, and τ_rec_ using [19]:

(3) $$L_{D}=\sqrt{D_{n}\tau_{\mathrm{rec}}}$$

**Figure 5:**
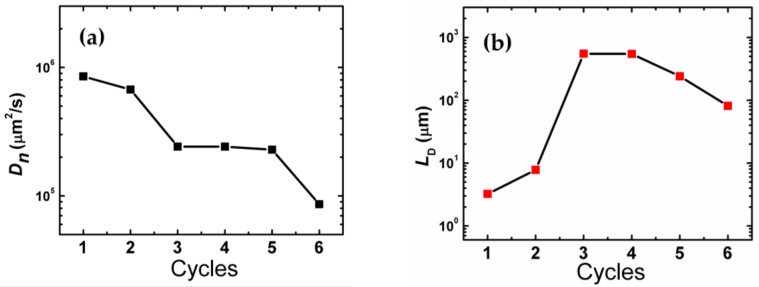

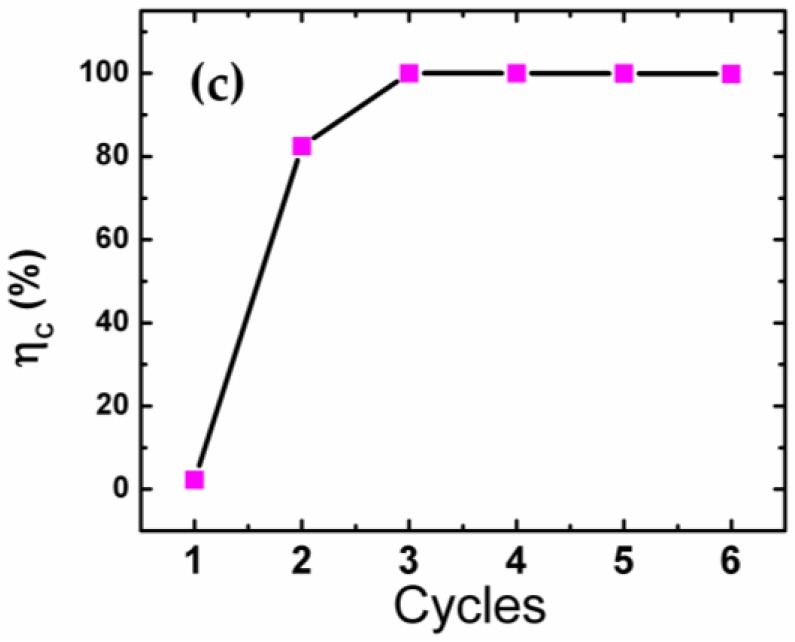
(**a**) Calculated diffusion coefficients and (**b**) Diffusion lengths for 1–6 SILAR cycles. (**c**) Charge collection efficiencies calculated for 1–6 SILAR cycles.

The results are plotted as a function of SILAR cycle number in Figure 5a,b, respectively. The decreasing value of *L*_D_ after four cycles implies that when more QD layers are added, the electron transport path is hindered. Though the QD layers improve the photocurrent, they introduce more disorder regarding the transport path. The charge collection efficiency, η_c_ (Figure 5c) was calculated using [20]

(4) $$\eta_{c}=1-\frac{\tau_{\mathrm{trs}}}{\tau_{\mathrm{red}}}$$

The transport time constants for the collection of injected electrons into the ZnO:TiO_2_ core shell film is on the order of microseconds, while the recombination time constants are significantly longer; on the order of seconds. The resulting charge collection efficiency is therefore very high for devices made using four or more cycles.

## 3. Discussion

Devices coated by less than 4 cycles showed weak photovoltage responses. This is likely due to insufficient coverage of CdSe QDs on the photoanodes required for photosensitization. At 1 cycle, we see an extremely fast recombination time on the order of 10 μsec. Though TiO_2_ nanoparticles ought to aid in the passivation of surface states on the ZnO NW surface [12], and thus decrease the recombination time, the fast recombination time observed here implies that the cell is still suffering considerable recombination loss. This may be attributed to the fact that the steady-state white light background and red light perturbation have a negligible effect on TiO_2_ and ZnO, which absorb in the near-ultraviolet (UV) range [21,22,23]. In other words, for three or less cycles charge generation is simply not occurring at any observable scale, and the true IMVS response may be clouded by the resistance-capacitance (RC) attenuation of the circuit at high frequencies [24,25].

At four or more cycles the photo-response of the cell increases significantly. The near semi-circular shape of the IMVS Nyquist plots in the complex plane indicate that the diffusion length, *L*_D_, has exceeded the photoanode film thickness, which is one criterion for efficient charge collection and injection [26]. Recombination times for cycles four through six decrease with increasing cycle number.

The decrease in τ_rec_ may be understood in the following way. As electrons migrate through the film to the transparent charge collecting fluorine-doped SnO (FTO) electrode, they undergo many trapping and de-trapping events, either through recombination with electron acceptors in the oxidized QDs or with redox electrolyte species [20]. Since recombination occurs primarily through trap-states than through conduction band states, the reduction of electron recombination times can be attributed to surface passivation induced by the increase of QD coverage [27,28,29]. This can suppress recombination pathways between the nanostructured photoanode and the electrolyte by acting as a radial energy barrier [10,16]. Electron injection into the conduction band of the ZnO:TiO_2_ nanostructured anode is therefore more encouraged in devices with more QD coverage and this is observed as a reduction of the recombination time, τ_rec_. As the QD coverage is increased, however, we would expect the creation of trap states at the QD-ZnO:TiO_2_ interface [6,30,31,32]. The slower recombination rate could also indicate that the increase of QDs is directly related to an increase in photogenerated charge carriers, whereby the extra carriers fill these interfacial trap states, reduce the trapping events and accelerate electron transport [19,33].

The Nyquist plot of the IMPS (Figure 3a) spectra yielded a single semicircle, which suggests electron transport was dominated by one type of process. Based on previous work [18], we attribute this process to surface trap-state assisted percolation due to the particle-like (rather than purely nanowire-like) morphology of the ZnO:TiO_2_ film. In our case, the nanostructured anode is comprised of single-crystalline ZnO nanowires fused under a film of TiO_2_ nanoparticles. The ZnO nanowire will have a higher conductance than the TiO_2_ nanoparticles and we would expect that electrons would seek out the path of least resistance through the film (i.e. the path with most ZnO) [16]. This kind of transport mechanism would then dominate over any direct transport down pure single-crystalline nanowires.

As the number of SILAR increases, τ_trs_ also increases. This phenomenon can be explained by considering the reduction of the conduction band edge difference between the QDs and the ZnO:TiO_2_ as the average QD size is increased with increasing cycle number. Since the electron injection efficiency depends on the number of unfilled surface traps, the loss of the conduction band difference would slow down the transfer process by creating more unfilled traps. When there are more unfilled traps, the number of trapping and de-trapping events increases and τ_trs_ also increases.

Regarding the rather low J_sc_’s, the reason may be related to the polymer electrolyte. The effect of this polysulfide polymer electrolyte on the Pt film integrity is not yet well known. Electrolytes that utilize S_x_^2−^ are known to interact with Pt. Sulfides can adsorb onto thin Pt surfaces causing a decrease in electrical conductivity [34]. Given that the Pt layer is rather thin (~200 nm), surface-adsorbed sulfide species may significantly influence the electro-catalytic activity of the cell. Pt-free counter-electrodes, such as Au or Cu_2_S, may be used in future studies to further enhance cell performance.

## 4. Materials and Methods

ZnO NWs were grown using a vapor-liquid-solid technique in a 1-in horizontal tube furnace using Zn foil (99.98%, Alfa Aesar, Ward Hill, MA, USA) and oxygen gas. TiO_2_ nanoparticles (NPs) (99.5%, Organics Aeroxide™ P25, ACROS Organics, Fair Lawn, NJ, USA) were spincoated from an ethanol solution onto the as-grown NWs and annealed for 8 hours at 850 ºC until the two materials were uniformly sintered.

CdSe quantum dots were deposited on the ZnO:TiO_2_ anode layer by a successive ionic layer adsorption and reaction (SILAR) method. 0.03 M Cd(NO_3_)_2_ (99% Fluka, Sigma-Aldrich, St. Louis, MO, USA) in ethanol was used as the cadmium ion source solution. The selenide ion source solution was prepared from SeO_2_ (99.9%, Sigma-Aldrich, St. Louis, MO, USA) and NaBH_4_ (99%, Sigma-Aldrich, St. Louis, MO, USA) also dissolved in ethanol. The ZnO:TiO_2_ anode was immersed in the Cd ionic solution, rinsed with pure ethanol and allowed to dry one minute. This was followed by immersion in the Se ionic solution, followed by the same rinsing and drying process. These six total steps comprised one cycle. Samples were coated with a final layer of ZnS (via immersion in a Zn^2+^ and S^2−^ ionic solution) to passivate the QD surface. All solution preparation and SILAR cycling was carried out in an argon filled glove box.

Solar cells were fabricated by adhering platinum coated fluorine-doped tin oxide (FTO) glass to the QD sensitized ZnO:TiO_2_ anode using a polymer electrolyte consisting of S/tetramethylammonium sulfate (S/TMAS) (99.5%, Sigma-Aldrich, St. Louis, MO, USA) redox additive to a poly(ethylene oxide)-Poly(vinylidene fluoride) (PEO-PVDF) (99%, Sigma-Aldrich, St. Louis, MO, USA) polymer base [35]. The anode was first coated with the electrolyte and heated in a drying oven at 80 °C for several minutes; until the electrolyte became viscous. The Pt-FTO glass was then pressed to the anode and baked at the same temperature for several hours to allow the electrolyte to dry completely and effectively glue the two electrodes together.

SEM images were obtained using FEI Quanta FEG 450 field-emission scanning electron microscope (FEI, Hillsboro, OR, USA). IPCE data was obtained using data was obtained using a monochromatic light source consisting of a 50 W tungsten halogen lamp and a monochromator (Newport Corporation, Irvine, CA, USA). The light beam was modulated by a chopper and a lock-in amplifier (Stanford Research SR830, Sunnyvale, CA, USA). Intensity modulated photocurrent and photovoltage (IMPS/VS) measurements were carried out under a constant white light background with a sinusoidal perturbation of red light (*λ* = 625 nm). The white light was supplied by a light-emitting diode (LED) array powered from a Yokogawa 7651 low-noise direct current (DC) supply (Newnan, GA, USA). The red light intensity was modulated by the output of a Stanford SR781 dynamic signal analyzer (Stanford Research SR830, Sunnyvale, CA, USA). The transfer function module of the SR780 detected the IMPS/VS signal, while a Stanford SR570 current preamplifier amplified the photocurrent response from the cell. The intensity of the red light output was maintained at less than 10% of the white light background intensity. The red light frequency was scanned from 0.1 Hz to 100 kHz. All measurements were made in the dark.

## 5. Conclusions

The electron transport properties of ZnO:TiO_2_ NW/NP CdSe QDSSCs were studied. The CdSe QDs were deposited by varying SILAR cycles numbers and coated with a ZnS passivation layer. Device performance was dependent on the cycle number. The photocurrent of the device increased with successive SILAR coatings and the highest photocurrent was observed for devices coated with 6 SILAR cycles. IMVS and IMPS measurements were carried out and the transport and recombination time constants and charge collection efficiencies were obtained. Both the recombination time and transport time were found to decrease with increasing SILAR cycles, indicating surface state passivation due to QD coverage. Due to its low toxicity and potential for optimizing the band alignment between the QD and electrode, ZnO:TiO_2_ systems may be an important active material in future QDSSC studies.
